# Enhancement of Volatile Fatty Acids Production from Food Waste by Mature Compost Addition

**DOI:** 10.3390/molecules24162986

**Published:** 2019-08-17

**Authors:** Yen-Keong Cheah, Joan Dosta, Joan Mata-Álvarez

**Affiliations:** 1 Department of Chemical Engineering and Analytical Chemistry, University of Barcelona, 08028 Barcelona, Spain; 2 Water Research Institute, University of Barcelona, 08001 Barcelona, Spain

**Keywords:** acetic acid, acidogenic fermentation, biorefinery, butyric acid, HRT, pH, propionic acid

## Abstract

Food waste (FW) collected from a university canteen was treated in acidogenic fermenters to produce volatile fatty acids (VFA) under biological pretreatment with mature compost. Batch assays working at pH 6 revealed an increment of 9.0%, 7.9%, and 4.1% (on COD basis) of VFA concentration when adding 2.5%, 3.5%, and 4.5% *w*/*w* of mature compost, respectively, even though the volatile solids (VS) concentration of food waste was lower in the tests with increasing doses of mature compost. For batch tests at pH 7, this VFA generation improvement was lower, even though enhanced COD solubilization was recorded. Operating in semi-continuous conditions at 35 °C, pH of 6, and hydraulic retention time (HRT) of 3.5 days, the addition of 2.5% *w*/*w* of mature compost led to a VFA concentration up to 51.2 ± 12.3% more (on VS basis) when compared to a reference reactor without compost addition. Moreover, the percentage of butyric acid on VS basis in the fermentation broth working at a pH of 6 increased from up to 12.2 ± 1.9% (0% compost addition) to up to 23.5 ± 2.7% (2.5% compost addition). The VFA production was not improved when a higher percentage of mature compost was used (3.5% instead of 2.5% *w*/*w*), and it slightly decreased when mature compost addition was lowered to 1.5% *w*/*w*. When working at a pH of 7 in the semi-continuous fermenters with the addition of 2.5% *w*/*w* mature compost at an HRT of 3.5 days, an improvement of 79% and 104% of the VFA concentration (on VS basis) were recorded as compared to fermenters working at a pH of 6 with 2.5% and 0% *w*/*w* of mature compost addition, respectively. At a pH of 7, higher production of propionic and valeric acids was found with respect to the reactor working at a pH of 6. The effect of pH on VFA generation was estimated to have greater contribution than that of only biological pretreatment using mature compost. At a pH of 7, the VFA yield was higher for the fermenter working with 2.5% *w*/*w* mature compost but at a pH of 7 and HRT of 5 days, the effect of mature compost on VFA production improvement was lower than that obtained at a pH of 6. Moreover, higher solubilization in terms of soluble chemical oxygen demand and total ammonium was detected when biological pretreatment using mature compost was applied at both a pH of 6 and a pH of 7, which indicates enhanced hydrolysis in both conditions.

## 1. Introduction

The raising concern about climate change and sustainability have led to an increasing awareness of resource utilization [1] and, under this context, the demand for energy and materials is now a big challenge in this century [2]. Urban organic wastes are known to contain a great variety of fermentable and biodegradable materials, which include biodegradable organic compounds such as sewage sludge (primary and secondary), food waste (FW), and organic fraction of municipal solid waste (OFMSW), among others. The acidogenic fermentation of urban organic wastes is gaining attention due to their high accessibility and possible process improvement. In 2012, approximately 90 million tons of FW was generated in all European countries [3]. This number indicates that a huge quantity of volatile fatty acids (VFA) could have been recovered through anaerobic acidogenic fermentation. VFA is a key commodity to produce biomaterials, such as polyhydroxyalkanoates, and biodegradable bioplastics, which currently have a growing market [4,5,6]. Other than that, VFA could be used as external carbon sources for biological nutrient removal (nitrogen and phosphorus) in wastewater treatment plants or to produce bioenergy [7,8,9]. To improve the biomaterials utilization from FW, such as PHA, it is of the utmost importance to maximize the production of VFA and different strategies are possible [10].

Hydrolysis is usually the rate limiting step for VFA production [11,12,13,14]. Hence, development of treatment methods to improve hydrolysis and solubilization of complex organic compounds has been investigated and it is still under research. Currently, there are a number of pre-treatment methods [15,16,17,18] to enhance the hydrolysis step, which is usually classified as physical, chemical, and biological. Physical pretreatments (thermal and mechanical) increase the disintegration of cell membranes or the specific surface area, which can provide better contact between substrate and microorganisms [19,20,21]. In chemical pretreatments, the external addition of chemicals (acids, alkalis, ozonation, etc.) will somehow increase the solubilization of the substrate [17,18,22]. Biological pretreatments are getting more attention for acidogenic fermentation, since they do not require reagent additions and do not imply high-energy demands. Among biological pretreatments, bioaugmentation is a promising approach to enhance VFA production instead of other organic compounds [10]. The simplest use of bioaugmentation is to add to the fermentation medium, which is a pre-adapted mixed culture. An example is the rumen addition that has been successfully used in anaerobic fermentation of corn stover [23] or anaerobic digestion of high lignocellulose grass silage [24]. Rumen addition typically requires an immediate use of it, since these bacteria are very sensitive and could become inactive after a few hours. Even so, the net positive output has attracted many researchers’ interest. Another approach that would overcome this problem is the use of mature compost, which is much more stable than rumen, but this biological pretreatment agent has been seldom reported in literature.

This paper focuses on the VFA production by adding mature compost to FW in acidogenic fermentation, to assess quantitatively the improvement yields and composition of VFA at different pHs and hydraulic retention time (HRT). To this end, batch and semi-continuous experiments were carried out for an operation period of 200 days.

## 2. Results and Discussion

### 2.1. Effect of Mature Compost Addition at pH of 6

**Figure 1:**
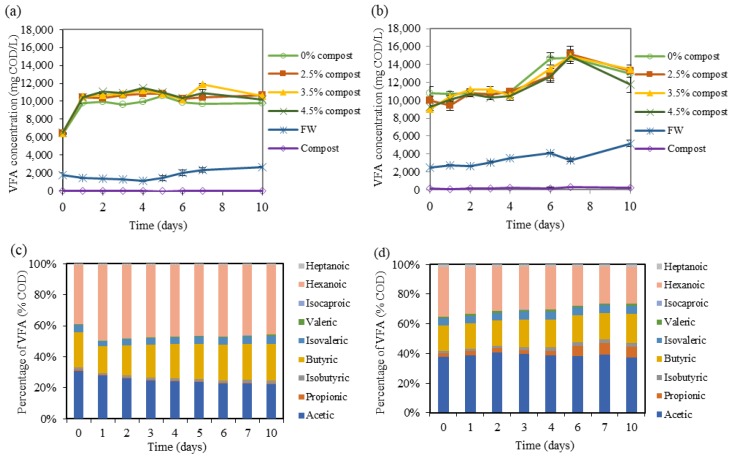
VFA concentration monitoring in a batch of acidogenic fermentation tests of FW at a pH of 6 (**a**) and a pH of 7 (**b**) with a different mature compost addition and evolution of the VFA distribution in the fermentation test with 2.5% *w*/*w* mature compost addition at a pH of 6 (**c**) and 7 (**d**).

Several batch tests were performed at a pH of 6.0 using mixtures 1:1 on the volatile solid (VS) basis of food waste and acidogenic fermentation inoculum of a digester treating FW [15] with different doses of mature compost (namely, 0%, 2.5%, 3.5%, and 4.5% *w*/*w*). Table 1 summarizes the main characteristics of these batch tests and Figure 1 shows the VFA production with different doses of mature compost. The results showed that, in a short-term period (10 days), an increment of 9.0%, 7.9%, and 4.1% of VFA concentration (on the COD basis) was observed when 2.5%, 3.5%, and 4.5% *w*/*w* of mature compost was added, respectively, as compared to the batch without compost addition (it should be noted that the quantity of food waste added in each batch decreased when the percentage of compost increased).

Within one day, the batch containing inoculum and FW (with or without compost) increased to reach a stable VFA concentration. Only small changes were recorded afterwards. This could be related to the easily fermentable organic material of fresh FW and the high activity of the fermentation inoculum. Bottles containing only FW could have inherent microorganisms of these substrates, but this biomass did not contribute much to the formation of VFA in acidogenic fermentation in 10 days. For the batch containing only mature compost, VFA production remained below 26 mg VFA/L in this batch. When analyzing the VFA distribution between acidogenic fermentation of FW with and without addition of mature compost, the difference was not clear, since every individual VFA changed in a similar way. The biggest change in VFA composition could be seen from day 0 to day 1, which was the moment when most VFA were produced and reached stable conditions afterward. Table 1 summarizes the percentage of the three main VFA products obtained at the 10th day of these batch assays, which are mainly composed by acetic, butyric, and hexanoic acids. Figure 1c shows the evolution of the VFA profile during the batch test at a pH of 6 where 2.5% *w*/*w* was added. It was observed that butyric and hexanoic acids concentrations had some changes in the first two days. In contrast, acetic acid was approximately stable during the first five days and remained at 22% COD of total VFA concentration at day 10. When considering the soluble chemical oxygen demand (sCOD) analyzed at the end of the batch tests (Table 1), higher solubilization of organic compounds could be observed when mature compost was added. Without addition of mature compost, only 27.4% increase in sCOD was observed while 46.8%, 44.5%, and 48.6% increase in sCOD was monitored when 2.5%, 3.5%, and 4.5 % mature compost was added. These indicated that, with the addition of mature compost, hydrolytic enzymes were introduced into the fermentation broth with the aim to increase the hydrolytic rate [25,26]. These results were also consistent with the study by Fdez.-Güelfo et al. [27] who tested the biological pretreatment of OFMSW using mature compost and reported that 2.5% *v*/*v* was enough to increase the sCOD by roughly 50%, which was the indicator of the solubilization yield in that study. When compost was added, FW was microbiologically solubilized prior to acidogenic fermentation [28]. In terms of total ammonium nitrogen (TAN), the percentage of solubilization of ammonium also increased abundantly.

**Figure 2:**
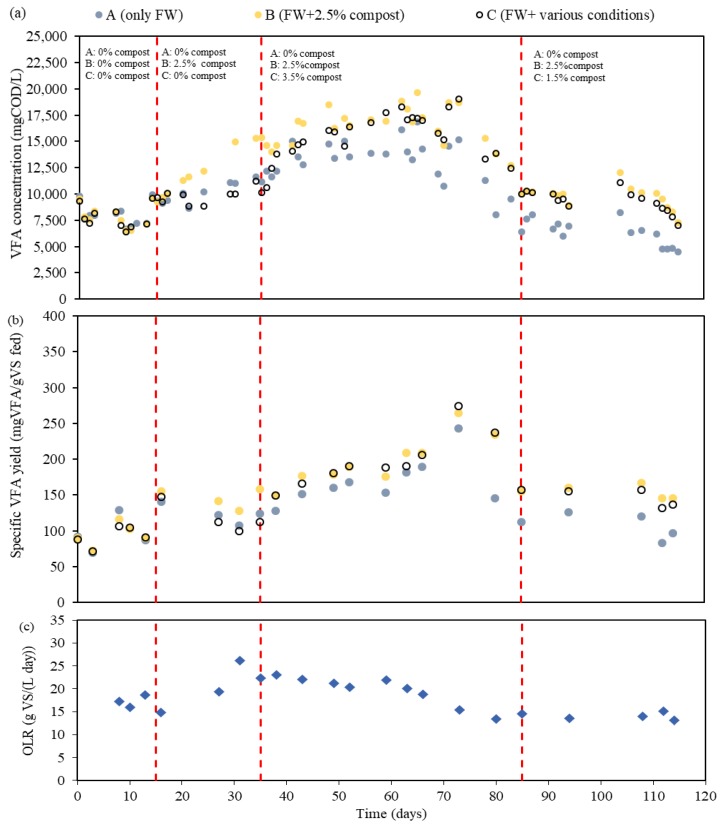
Evolution of (**a**) VFA concentration of the fermentation broth, (**b**) specific VFA production, and (**c**) applied organic loading rate (OLR) when treating food waste (FW) with several percentages of mature compost at a pH of 6 in fermenters A, B, and C.

**Figure 3:**
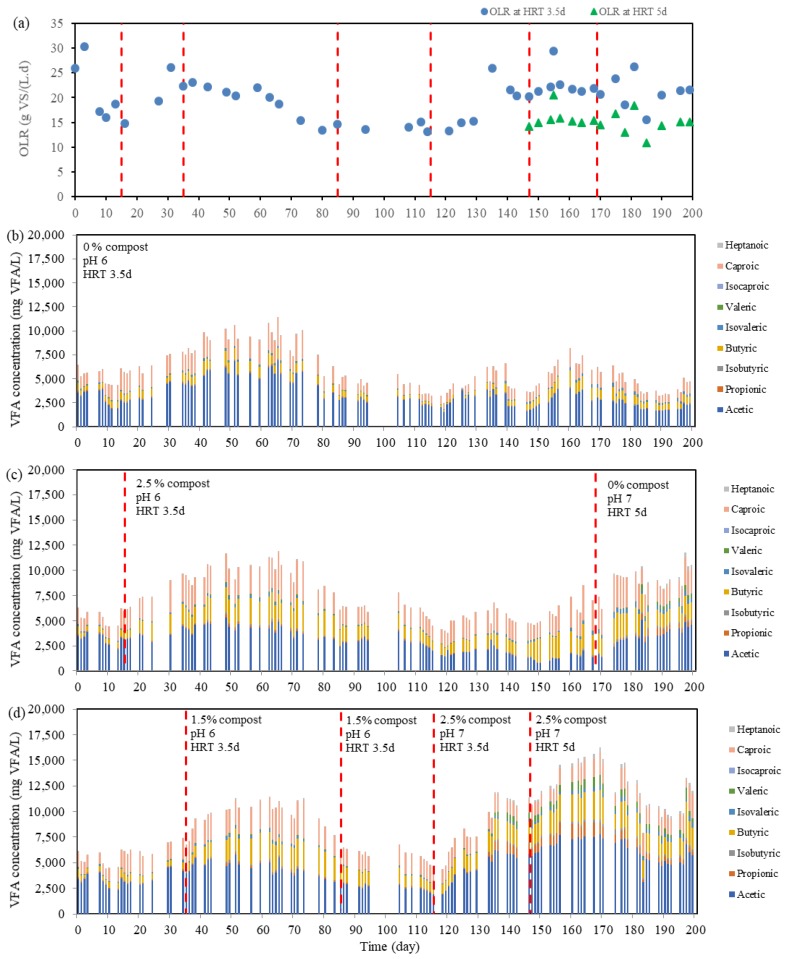
OLR (**a**) and individual VFA concentration in the whole experimental period of fermenters A (**b**), B (**c**), and C (**d**).

To study the effects in long-term conditions, three semi-continuous reactors (A, B, and C) were put into operation at a pH of 6 without compost addition working under a retention time of 3.5 days. After 15 days of operation with approximately the same specific VFA production in terms of g VFA/g VS fed, several doses of mature compost were added to the digesters. Table 2 summarizes the different periods of FW collection carried out where it is observed that fermenter A (fed with only FW) remained as a reference reactor, fermenter B worked always with 2.5% (on dry weight) of mature compost (mixed in the influent FW), and fermenter C worked under different conditions of mature compost addition. Figure 2 monitors the VFA concentration profiles (in terms of mg COD_VFA_/L), the VFA yield (expressed in terms of g VFA/g VS_fed_), and the Organic Loading Rate (OLR) applied (in terms of g VS/(L day)) during Phase 1. On the other hand, Table 3 shows the main characteristics of the effluent fermentation broth obtained in each period and Figure 3 shows the monitoring of the individual VFA during the whole experimental period.

When 2.5% *w*/*w* of mature compost was mixed with FW as feeding of fermenter B, an increase of VFA production in comparison to the reference fermenter was observed. The VFA yield reached its maximum 0.27 gVFA/gVS on day 73 after the addition of 2.5% *w*/*w* of mature compost, with an average improvement in VFA yield of 30.6 ± 16.7% (VS basis) in that the FW collection period with respect to the reference reactor. When fermenter C was fed with FW and 3.5% *w*/*w* of mature compost (days 35–84), the production of VFA on average, was 29.8 ± 14.4% more (VS basis) than that of the reference fermenter. Therefore, it yielded similar results than the fermenter working with 2.5% *w*/*w* mature compost. To further understand the relationship between the percentage of mature compost and VFA production under these operating conditions, the percentage of mature compost in the feeding was decreased from 3.5 to 1.5% *w*/*w* in fermenter C. When the percentage of mature compost was lowered, the VFA yield slightly decreased with respect to fermenter with 2.5% *w*/*w* mature compost. Therefore, in both long-term and short-term periods, with the presence of mature compost, VFA production could be limitedly boosted up in the beginning and maintained its dominance during the experiment, without considering the quantity of doses. Generally, higher solubilization expressed in terms of sCOD was detected in the fermenters working with mature compost addition (see Table 3), which is also consistent with a higher NH_4_^+^-N concentration in the fermentation broth. This might be due to the mature compost, which contains a variety of microorganisms, aerobic and anaerobic, with hydrolytic enzymes, which are capable of improving the solubilization of biodegradable organic matter [27]. Moreover, butyric acid percentage on a VS basis in the fermentation broth was enhanced due to mature compost addition at a pH of 6, which increases from up to 12.2 ± 1.9% (without compost addition) to up to 23.5 ± 2.7% (2.5% *w*/*w* compost addition).

Aside from the changes of mature compost percentage, it can be clearly seen that the VFA concentration of the reference fermenter was not constant, since the influent feedstock was not exactly the same in every collection and also the OLR varied from one period to the other. The mature compost addition resulted in a higher VFA yield with respect to the reference reactor in those periods when the OLR was lower. These results also reveal the current situation of VFA produced through acidogenic fermentation of real FW obtained directly from local restaurants, canteens, or households. Therefore, the fluctuations emerged during this experiment could be caused by the continuous changing of real FW every two to three weeks.

### 2.2. Effect of Mature Compost Addition at a pH of 7

Figure 1b shows the evolution of VFA concentration in batch assays treating FW under 35 °C and a pH of 7. Table 4 summarizes the main characteristics of these batch tests. From the results of batch assays, it could be observed that the VFA concentration steadily increased but did not show a large increment from day 0 to day 4. More VFA started to be produced from day 4 onward and reached the maximum observed VFA concentration on day 7. Batch tests with 2.5%, 3.5%, and 4.5% mature compost were able to reach 15.2 ± 0.9, 14.9 ± 0.8, and 14.9 ± 0.6 gCOD_VFA_/L, respectively, at day 7. In this batch test, the bottles containing 0% mature compost followed the VFA production trend of those containing various percentages of mature compost ranging from 2.5% to 4.5% and their difference was relatively small (it should be noted that the quantity of food waste added in each batch decreased when the percentage of compost increased). A VFA concentration below 175 mg/L was detected in the control assay where only mature compost was added. The evolution of the distribution of individual VFA for the batch test with 2.5% compost (*w*/*w*) at a pH of 7 is shown in Figure 1d, where it is observed that the hexanoic acid percentage decreased with time while propionic and butyric acids increased. The day when the maximum VFA concentration was recorded (day 7) at a pH of 7 by adding 2.5% *w*/*w* compost. The main VFA constituent was acetic acid, accounting for approximately 5.99 gCOD/L, which was followed by hexanoic (3.79 gCOD/L), butyric (2.71 gCOD/L), propionic (1.16 gCOD/L), and isovaleric acid (0.78 gCOD/L). A similar proportion of VFA was reported in the experiment carried out by Kumar and Mohan [29] treating vegetable waste, considering only short chain carboxylic acids (C_2_–C_5_). Moreover, these authors [29] obtained 2.6% and 7.0% propionic acid in the fermentation broth at a pH of 6 and a pH of 7, respectively, which is in line with the results obtained in this study, where a significant increment of propionic acid production was observed from day 4 to day 6 when working under neutral pH and mature compost addition (see Figure 1d). These results brought to the conclusion that acidogenic fermentation at neutral pH changed the metabolic pathway, which leads to the production of propionic acid as compared to partially acidic pH. To define the state of acidogenic fermentation, some researchers used the degree of acidification [29,30,31], which represents the bioconversion of substrate into short chain carboxylic acids (VFA in this study). In a short-term period, an increase in sCOD and total ammonium nitrogen (TAN) can be observed (see Table 4). Higher sCOD after acidogenic fermentation proved the hydrolysis and solubilization occurred in this fermentation process. Without adding mature compost, sCOD could raise from 40.6 gCOD/L to 48.5 gCOD/L and the addition of 3.5% mature compost led to the highest increment, which was 25.4%, from 39.1 gCOD/L to 49.1gCOD/L. This value shrunk when the mature compost addition was increased from 3.5% to 4.5%. Fdez.-Güelfo et al. [27] compared the effect of solubilization with different doses of mature compost varying between 2.5% to 10% (*v*/*v*) and a big difference in the increment of sCOD was found between 2.5% and 5% mature compost, where 2.5% yielded the highest and 5% was only half of that. In their experiment, the lowest doses produced the highest increase of sCOD, which might be due to higher consumption of solubilized organic compounds when metabolic activity increases. On the other hand, TAN was directly proportional to the mature compost added without addition of mature compost. TAN was able to raise 60% respect to its initial value. This number was even higher when a different percentage of mature compost was added.

**Figure 4:**
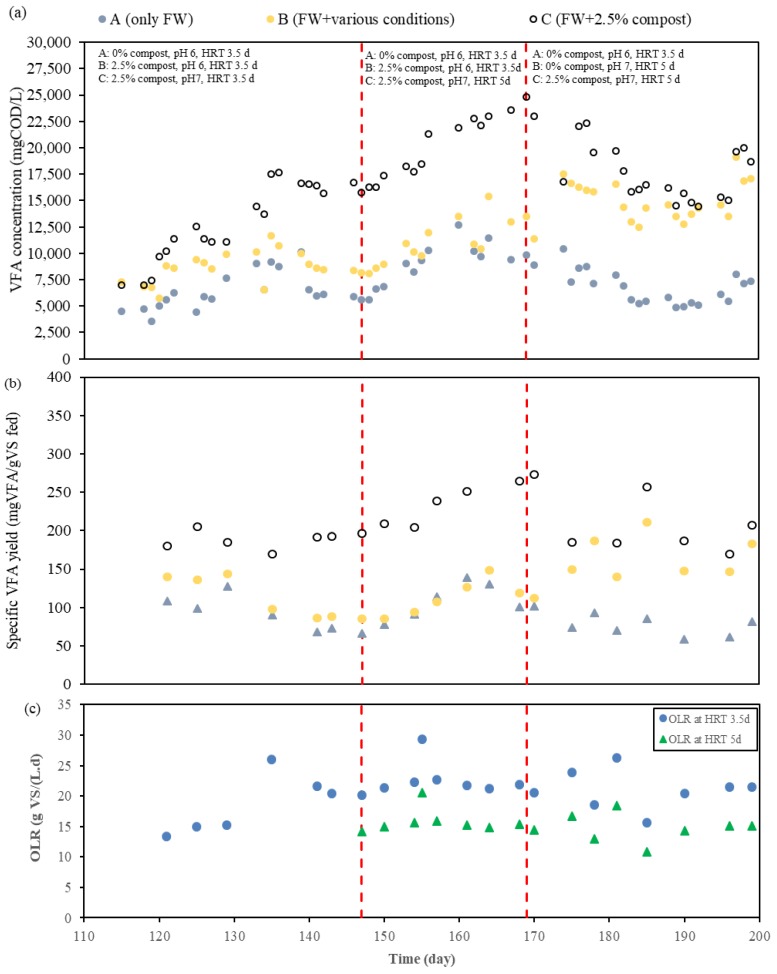
Evolution of (**a**) VFA concentration of the fermentation broth, (**b**) specific VFA production, and (**c**) applied OLR when treating FW with several percentages of mature compost at a pH of 6 and pH of 7 in fermenters A, B, and C.

Regarding the semi-continuous operation of the acidogenic fermenters, at the beginning of Phase 2, a change from pH 6 to pH 7 was applied to fermenter C. As shown in Figure 4, after changing to pH 7, the microorganisms responded quickly and the total VFA concentration of fermenter C was boosted five days later. A stepwise increment of VFA concentration from day 120 to 125 and from day 130 to 135 was related to a sudden increase in OLR, due to different FW collection periods. However, VFA production in fermenters A and B followed the same trends as in the previous period since they remained working at a pH of 6. FW is known by its heterogeneous composition in terms of carbohydrates, proteins, and lipids [32], and the randomly collected food waste from a University canteen leads to added complexity related to the different composition of this waste. Mixed microbial cultures (MMC) make VFA production possible through different metabolic pathways, which depend on the type of substrates (carbohydrates, proteins, and lipids) [33], among other factors. Nevertheless, there could exist some hindrances to retard or even stop the VFA generation by MMC, where one of them is steric hindrance from a residual extracellular polymeric substance (EPS) polymeric network [34]. EPS molecules result in the formation of a tight extracellular matrix (ECM) covering cells that constitute barriers, which will prevent the penetration of hydrolytic enzymes during digestion [35]. A large gap between fermenters working at a pH of 6 (A and B) and a pH of 7 (C) can be noted by the end of the first stage of Phase 2. In this phase, the biological pretreatment at a pH of 6 contributed to 11% to 25% improvement on VFA generation (on VS basis) with respect to the reference reactor at a pH of 6. The combined effect of regulating the pH to 7 while dosing mature compost yielded a net positive enhancement of VFA production. At a pH of 7 with the addition of 2.5% mature compost with an HRT of 3.5 days, an improvement of 79% and 104% of the VFA concentration (on VS basis) were recorded during this phase (days 133–145) as compared to fermenters B and A, respectively. From these comparisons, the effect of pH on VFA generation was estimated to have greater contribution than that of only biological pretreatment using mature compost. The regulation of pH to a value near 7 enhanced the solubilization of FW and resulted in having more sCOD and NH_4_^+^-N, as summarized in Table 3, and a concomitant higher VFA production. Moreover, the VFA profile at a pH of 7 with mature compost addition was different from that obtained at a pH of 6 (as stated in Table 3 and Figure 3). A rise in propionic, valeric, and isovaleric acid production was observed.

In addition to working at a pH of 7, the HRT of Fermenter C was increased from 3.5 to 5 days. At longer HRT, more time was given to bacteria to undergo decomposition of long chain organic molecules, especially the rate-limiting hydrolysis step. Although it could be possible to achieve higher VFA production, risk of interference of methanogenic activities should also be taken into account [32]. Figure 3 and Figure 4 show the VFA concentration of the semi-continuous operation of this fermenter. When HRT was increased to 5 days in fermenter C, VFA concentration in the fermentation broth increased linearly from 15.8 gCOD/L to 24.8 gCOD/L and remained within these limits in the third stage, with changes related mainly to OLR and the collected FW. In this case, it was unclear whether extending HRT from 3.5 days to 5 days had led to higher VFA yield, since, even though, the VFA yield increased during this period in Fermenter C, the VFA yield improvement with respect to the reference reactor (fermenter A) was in the same range. Kuruti et al. [36] performed some experiments to examine the optimal HRT for acidogenic fermentation and found out that, irrespective of the pH, batch fermentation broths were stabilized on day 4, 7, and 5 when working with glucose, cattle manure, and poultry litter, respectively. Moretto et al. [37] also suggested to consider HRT of 6 days to achieve best performance of process yield and maximum VFA level through the results of their experiments when treating a mixture of squeezed OFMSW and thickened waste activated sludge at a pH of 9 and mesophilic temperature (37 °C). In this study, the mesophilic fermenter containing 2.5% mature compost and operating at a pH of 7 produced the highest quantity of VFA when compared with the other two in operation.

Regarding the VFA profile (on VS basis) in this fermenter, acetic, propionic, butyric, isobutyric, valeric, and isovaleric acids accounted for the 48.7 ± 3.0%, 4.6 ± 0.9%, 18.4 ± 2.8%, 1.67 ± 0.19%, 4.63 ± 1.49%, and 3.44 ± 0.65%, respectively, of the total amount of VFA produced during the last stage of the experiment in accordance to the last FW collection period. Furthermore, in the last stage, the working pH of Fermenter B was regulated to 7, the retention time was extended to 5 days, and the mature compost addition was stopped. The net VFA production after making these modifications was positive, and a pH regulation to 7, together with increasing HRT, had greater effects on VFA generation. Comparing VFA production from fermenter B and C, VFA yield was higher for the fermenter working with mature compost but it was not possible to conclude that, at a pH of 7 and HRT of 5 days, the effect of mature compost on VFA production was as significant as when working at a pH of 6 (Phase 1). However, COD solubilization and NH_4_^+^-N release was higher when adding mature compost at a pH of 7 and HRT 5 days, which led to a lower ratio of VFA concentration with respect to soluble COD for the fermenter with mature compost addition, as stated in Table 3.

## 3. Materials and Methods

### 3.1. Substrate and Inoculum

The FW was collected from a university canteen every two weeks, approximately. Although the composition changed between collection periods, it mainly contained pasta, potato, meat, rice, noodles, bread, vegetables, and fruit peels. Once collected, FW was shredded (Bosch, Spain, MMB66G5M) and mixed with deionized water in a proportion of approximately 1:2 by volume, to obtain a concentrated feedstock of FW. This FW was stored in a refrigerator chamber at 4 °C until its usage. When the feedstock was needed for acidogenic fermentation, this substrate was diluted with deionized water to control the contents of total solids (TS, 4.45–7.57%) and volatile solids (VS, 3.78–6.43 %) so that their values were within a certain range during the experiments. The characterization of FW collected in different periods is shown in Table 5. The mature compost was collected from a full-scale mechanical-biological treatment (MBT) plant in the metropolitan area of Barcelona and came from tunnel composting of OFMSW and park and garden waste. The collected compost contained 52% of TS and 27% of VS. Once it was collected, compost was stored in a closed vessel to preserve its original moisture contents. Unlike FW, the mature compost was collected in one single time. Therefore, the characteristics of the mature compost were constant throughout the experiments. The inoculum used was effluent of acidogenic fermenter treating FW at a pH of 6 and hydraulic retention time (HRT) of 3.5 days from a previous work [15].

### 3.2. Experimental Set-Up

#### 3.2.1. Batch Assays: FW with Mature Compost

Discontinuous assays were performed in serum bottles of a 200-mL effective volume with different mature compost doses (0, 2.5, 3.5, and 4.5% *w*/*w*) to study the effects of mature compost on VFA production and their individual VFA profile. Serum bottles were filled with inoculum and substrate according to their volatile solids content, to obtain an inoculum to substrate ratio (ISR) of 1:1 by weight. Corresponding mature compost doses (0, 2.5, 3.5, and 4.5% *w*/*w*) were then added. Blank samples were prepared as a control, including inoculum, substrate, and mature compost (three times the weight for 4.5% *w*/*w* and filling with deionized water). In different periods of time, two batches were carried out operating at a pH of 6 and a pH of 7 by using sodium bicarbonate (NaHCO_3_) and hydrochloric acid (10 M HCl) for pH adjustment. Before the bottles were sealed with a stopper containing PTFE/Butyl septum, nitrogen gas was flushed through the headspace to remove residual air. These bottles were located inside an incubator (Memmert GmbH + Co. KG, Schwabach, Germany, Pass-through ovens UF750) working at a mesophilic temperature (35 °C). Furthermore, 2.5 mL of the sample were taken and centrifuged at 4000 rpm for 15 min. The supernatant was filtered by a 0.45-μm syringe filter. Part of this filtered sample was used for soluble chemical oxygen demand (sCOD) and total ammonium nitrogen (TAN) analysis in day 0 and day 7 while the other part of the filtered sample was prepared for VFA analysis where this operation was performed daily from day 0 to 7, and, on day 10, to check the stability of VFA production. The pH measurements were performed in the beginning (day 0) and the end of the batch test (day 10), and two selected days in between (day 3 and 7). In these days when the measurement was carried out, pH was adjusted to their initial pre-set values in order to avoid different behaviors on VFA production due to pH fluctuation. Analyses were performed in duplicate for the standard deviation calculation.

#### 3.2.2. Semi Continuous Operation

For this study, three jacketed lab-scale reactors with an effective working volume of 4.5 L and mechanically stirred (using IKA-Werke, IKA Werke GmbH & Co. KG, Staufen, Germany, RW 16 basic functioning at approximately 150 rpm) were used as acidogenic fermenters at mesophilic conditions (35 °C) working with FW. These fermenters were operated at HRT of 3.5 days and the equivalent quantity of substrate (FW) was fed manually once per day (fed-batch culture). During feeding and draw-off operations, a minimum amount of nitrogen gas was flushed through the headspace of fermenters to avoid entering air.

At the beginning of the experiment, the three fermenters (A, B, and C) were fed with FW for 15 days. At this moment, all fermenters were working at HRT of 3.5 days and a pH of 6, and NaHCO_3_ was used to increase alkalinity of the fermentation broth and to adjust pH to the pre-set value. The whole experiment was carried out under mesophilic conditions (35 °C). Fermenter A remained as the reference reactor and was always fed with FW without adding mature compost.

To analyze the long-term effect of biological pretreatment using mature compost on the acidogenic fermenter, fermenter B was fed with a mixture of FW and compost, which represented the 2.5% *w*/*w*. In fermenter C, different doses of mature compost were added to test the effect of the quantity of mature compost addition. Later, in addition, to study the effects of biological pretreatment, pH and HRT were taken into consideration. Table 2 summarizes the compost addition, working pH, and HRT applied in each acidogenic fermenter. As stated in this Table, to evaluate the fermenters’ performance at a different pH and HRT conditions, the reference fermenter (A) worked at a pH of 6, HRT 3.5 days, and without mature compost addition and the other fermenters could work at a pH of 6 or 7, at different HRT of 3.5 or 5 days, and at different mature compost dosages (up to 3.5% *w*/*w*) depending on the parameter to be studied. Samples were taken daily on weekdays for continuous assessment of VFA and effluent pH, and other characterizations were analyzed two to three times per week.

### 3.3. Analytical Methods

The analysis of soluble COD, total solids (TS), and volatile solids (VS) were performed in accordance to the Standards Methods for the Examination of Water and Wastewater [38]. For the determination of the total ammonium nitrogen (TAN) concentration, the sample was centrifuged at 4000 rpm for 15 min, the supernatant was filtered through a 0.45 μm-pore size regenerated cellulose syringe filter. Samples were properly diluted to have the TAN range between 1 to 100 ppm and were analyzed with an ammonium ion selective electrode (Thermo Scientific, Beverly, MA, USA, Orion 9512HPBNWP). The pH was determined using a Crison pH electrode (pH series 52–04). Filtered samples were acidified with 85% phosphoric acid for VFA concentration analysis. Individual VFA including acetic, propionic, isobutyric, butyric, isovaleric, valeric, isocaproic, caproic, and heptanoic acids were determined by gas chromatography (Shimadzu GC 2010 plus) (Shimadzu Corporation, Kyoto, Japan) equipped with a capillary column (Nukol™, 15 m × 0.53 mm × 0.5 μm) and flame ionization detector (FID) (Shimadzu Corporation, Kyoto, Japan). The initial temperature of capillary column was 80 °C and increased at 10 °C/min to 110 °C, from 110 °C to 145 °C at 15 °C/min, which was followed by an increment of 20 °C/min to reach 190 °C. The temperature was 280 °C and 300 °C to the injector and detector, respectively. The carrier gas was helium, the fuel gas was hydrogen, and the oxidizing gas was synthetic air. Hence, individual VFA were detected by the programmed method for this gas chromatograph.

## 4. Conclusions

In this study, FW collected from a university canteen was treated in acidogenic fermenters to produce volatile fatty acids (VFA) under biological pretreatment with mature compost. Batch assays working at a pH of 6 revealed an increment of 9.0%, 7.9%, and 4.1% (on COD basis) of VFA concentration when adding 2.5%, 3.5%, and 4.5% *w*/*w* of mature compost, respectively, even though the VS concentration of food waste was lower in the tests with increasing doses of mature compost. When increasing the operating pH to 7, batch tests also revealed higher COD solubilization due to mature compost addition, even though it did not significantly impact enhanced VFA concentration in the fermentation effluent.

The addition of 2.5% *w*/*w* of mature compost to a semi-continuous acidogenic fermenter treating FW at mesophilic conditions (35 °C), pH of 6, and HRT of 3.5 days led to an improvement of the volatile fatty acids (VFA) yield up to 51.2 ± 12.3% (on VFA basis) when compared to the reference reactor working without mature compost addition. The mature compost dosage at a pH of 6 resulted in a higher percentage of butyric acid on a VS basis in the fermentation broth, which increases from up to 12.2 ± 1.9% (0% compost addition) to up to 23.5 ± 2.7% (2.5% *w*/*w* compost addition). Moreover, a higher sCOD content and a higher NH_4_^+^-N concentration was monitored in reactors working with mature compost, as a result of an enhanced hydrolytic activity. Considering the reactor working at a pH of 6 with 2.5% *w*/*w* addition, a higher compost addition of 3.5% *w*/*w* did not lead to higher VFA yield, while a slightly lower VFA production was recorded when working with 1.5% *w*/*w*.

When pH was changed from a pH of 6 to a pH of 7, VFA production was boosted and a higher production of propionic and valeric acids was recorded with respect to the reactor working at a pH of 6. In the semi-continuous fermenters with addition of 2.5% *w*/*w* mature compost at a pH of 7 with an HRT of 3.5 days, an improvement of 79% and 104% of the VFA concentration (on VS basis) were recorded as compared to fermenters working at a pH of 6 with 2.5 and 0% *w*/*w* of mature compost addition, respectively. Therefore, the effect of pH on VFA generation was estimated to have greater contribution than that of only biological pretreatment using mature compost. At a pH of 7, the VFA yield was higher for the fermenter working with mature compost but it was not possible to conclude that at a pH of 7 and HRT of 5 days, the effect of mature compost on VFA production was as significant as in a pH of 6. However, COD solubilization and NH_4_^+^-N release was higher when adding mature compost at a pH of 7 and HRT 5 days, which led to a lower ratio of VFA concentration with respect to soluble COD for the fermenter with mature compost addition.

## Figures and Tables

**Table 1:** Main characteristics of the batch tests of food waste using mature compost at a pH of 6.

Parameter	Units	0%	2.5%	3.5%	4.5%	FW Only	Compost Only
Food Waste weight	g	84.75	82.63	81.79	80.94	200	-
% VS of Food Waste in the mixture	%	50	44	41	39	100	-
Compost weight	g	-	5	7	9	-	27
% VS of Compost in the mixture	%	-	13	17	21	-	100
Inoculum weight	g	115.25	112.36	111.21	110.06	-	-
% VS of Inoculum in the mixture	%	50	44	41	39	-	-
Initial VS content	%	5.36	5.77	6.24	6.52	6.81	1.18
Initial soluble COD	g COD/L	36.95	34.64	34.56	35.14	41.22	5.21
Soluble COD at day 10	g COD/L	47.09	50.87	49.95	52.22	55.51	5.18
Initial NH_4_^+^-N	mg NH_4_^+^-N/L	355	345	353	339	47	1
NH_4_^+^-N at day 10	mg NH_4_^+^-N/L	1027	1058	1033	1058	458	16
VFA concentration and distribution at day 10							
VFA concentration	g COD/L	9.82	10.70	10.59	10.22	2.68	0.03
Acetic Acid	% COD	21.5	22.3	22.0	22.7	79.0	85.2
Propionic Acid	% COD	0.5	0.6	0.7	0.6	4.8	-
Isobutyric Acid	% COD	2.0	1.8	1.8	1.9	2.1	-
Butyric Acid	% COD	21.0	23.6	23.8	24.5	4.9	7.1
Isovaleric Acid	% COD	5.7	5.3	5.0	5.3	0.7	-
Valeric Acid	% COD	0.5	0.7	0.7	0.7	2.1	-
Isocaproic Acid	% COD	0.2	0.2	0.2	0.2	0.5	-
Hexanoic Acid	% COD	47.4	44.6	44.9	43.1	5.2	7.7
Heptanoic Acid	% COD	1.0	0.9	0.8	0.9	0.7	-

**Table 2:** Summary of the working conditions for the three semi-continuous mesophilic fermenters in the periods tested.

Fermenter	Phase	1				2		
	Stage	1	2	3	4	1	2	3
	Period (Days)	0–14	15–34	35–84	85–114	115–146	147–168	168–198
A	pH	6	6	6	6	6	6	6
	HRT (days)	3.5	3.5	3.5	3.5	3.5	3.5	3.5
	Compost (% *w*/*w*)	-	-	-	-	-	-	-
B	pH	6	6	6	6	6	6	7
	HRT (days)	3.5	3.5	3.5	3.5	3.5	3.5	5
	Compost (% *w*/*w*)	-	2.5	2.5	2.5	2.5	2.5	-
C	pH	6	6	6	6	7	7	7
	HRT (days)	3.5	3.5	3.5	3.5	3.5	5	5
	Compost (% *w*/*w*)	-	-	3.5	1.5	2.5	2.5	2.5

**Table 3:** Summary of the main characteristics of the effluent fermentation broth for each fermenter (A, B, and C) depending on the food waste collection period.

	Parameters	Units	FW1	FW2	FW3	FW4	FW5	FW6	FW7	FW8	FW9	FW10	FW11
	Period	Days	1–20	21–40	41–57	58–72	73–96	97–114	115–132	133–145	146–155	156–180	181–198
FERMENTER A	pH	-	5.74 ± 0.33	5.83 ± 0.27	5.91 ± 0.16	5.95 ± 0.20	5.91 ± 0.17	5.92 ± 0.21	5.90 ± 0.35	5.95 ± 0.27	5.90 ± 0.28	5.99 ± 0.13	6.05 ± 0.17
	VFA	g/L	5.54 ± 0.68	7.34 ± 0.85	9.60 ± 0.57	9.42 ± 1.26	5.67 ± 1.74	4.15 ± 0.79	3.85 ± 0.78	5.39 ± 1.09	4.47 ± 1.03	6.18 ± 1.00	3.85 ± 0.64
	sCOD	g/L	46.3 ± 6.4	48.1 ± 3.7	49.9 ± 2.5	48.5 ± 1.6	43.4 ± 3.5	46.0 ± 2.4	47.3 ± 5.6	45.7 ± 1.6	28.8 ± 5.9	35.2 ± 6.8	27.2 ± 3.69
	COD_VFA_/sCOD	%	20.4 ± 3.4	22.3 ± 3.1	26.8 ± 2.4	29.5 ± 1.8	21.6 ± 9.2	12.1 ± 2.7	12.2 ± 4.5	16.1 ± 3.7	25.6 ± 3.9	27.6 ± 3.5	19.6 ± 2.0
	NH_4_^+^-N	g NH_4_^+^-N/L	0.62 ± 0.27	0.52 ± 0.04	0.67 ± 0.04	0.39 ± 0.21	0.21 ± 0.06	0.25 ± 0.07	-	0.46 ± 0.07	0.59 ±0.02	0.81 ± 0.12	0.95 ± 0.15
	VFA yield	mgVFA_eff_/gVS_inf_	103.7 ± 29.9	120.5 ± 8.9	159.8 ± 8.1	174.8 ± 19.0	156.8 ± 59.4	100.4 ± 18.8	111.6 ± 14.8	78.7 ± 15.7	78.4 ± 12.8	107.4 ± 22.4	68.7 ± 12.2
	Acetic acid	% VS	53.7 ± 9.2	55.8 ± 4.7	60.3 ± 3.7	59.0 ± 2.8	58.3 ± 3.4	64.1 ± 3.6	68.9 ± 9.9	57.8 ± 5.6	51.0 ± 4.0	49.9 ± 4.1	49.6 ± 3.1
	Propionic acid	% VS	1.79 ± 0.25	1.31 ± 0.15	1.22 ± 0.11	0.96 ± 0.57	1.48 ± 0.54	1.64 ± 0.57	0.57 ± 0.55	0.77 ± 0.28	0.79 ± 0.26	0.79 ± 0.19	0.95 ± 0.20
	Isobutyric acid	% VS	0.82 ± 0.12	0.71 ± 0.06	0.72 ± 0.10	0.70 ± 0.09	0.58 ± 0.14	0.75 ± 0.06	0.38 ± 0.34	0.53 ± 0.24	1.05 ± 0.38	1.17 ± 0.29	1.59 ± 0.20
	Butyric acid	% VS	11.6 ± 3.0	11.2 ± 2.4	12.2 ± 1.9	10.7 ± 1.4	11.4 ± 1.0	7.4 ± 1.4	6.2 ± 2.6	11.2 ± 1.9	17.1 ± 0.9	17.2 ± 2.7	17.3 ± 1.8
	Isovaleric acid	% VS	1.82 ± 0.38	1.75 ± 0.28	1.86 ± 0.31	1.94 ± 0.32	1.27 ± 0.24	1.29 ± 0.29	0.91 ± 0.44	2.03 ± 0.72	3.36 ± 1.50	4.09 ± 1.17	5.38 ± 0.99
	Valeric acid	% VS	0.83 ± 0.12	0.58 ± 0.10	0.56 ± 0.05	0.55 ± 0.07	0.72 ± 0.20	0.91 ± 0.10	0.46 ± 0.45	0.49 ± 0.33	0.49 ± 0.25	0.63 ± 0.16	0.79 ± 0.31
FERMENTER B	pH	-	5.72 ± 0.33	6.01 ± 0.21	6.14 ± 0.16	6.02 ± 0.12	6.01 ± 0.10	6.14 ± 0.20	6.18 ± 0.18	6.05 ± 0.11	5.94 ± 0.15	6.47 ± 0.54	7.00 ± 0.21
	VFA	g/L	5.67 ± 0.83	8.74 ± 0.91	10.30 ± 0.81	10.60 ± 0.95	7.22 ± 1.51	6.21 ± 0.89	4.81 ± 0.70	6.15 ± 1.51	4.96 ± 0.42	7.88 ± 1.50	9.20 ± 1.09
	sCOD	g/L	46.1 ± 6.3	49.0 ± 0.7	49.1 ± 7.3	47.6 ± 7.4	49.1 ± 5.6	47.4 ± 3.2	46.3 ± 6.7	44.8 ± 1.3	30.3 ± 3.0	51.4 ± 20.3	40.3 ± 0.5
	COD_VFA_/sCOD	%	20.8 ± 4.4	27.4 ± 3.8	34.6 ± 6.2	39.2 ± 7.1	25.7 ± 7.0	19.3 ± 1.4	18.8 ± 2.9	21.5 ± 2.6	29.4 ± 1.1	28.6 ± 10.1	34.4 ± 1.1
	NH_4_^+^-N	g NH_4_^+^-N/L	0.63 ± 0.28	0.54 ± 0.02	0.83 ± 0.18	0.55 ± 0.31	0.28 ± 0.07	0.36 ± 0.08	-	0.56 ± 0.22	0.56 ± 0.27	0.88 ± 0.24	1.31 ± 0.01
	VFA yield	mgVFA_eff_/gVS_inf_	104.4 ± 29.0	144.6 ± 12.8	181.5 ± 7.1	197.9 ± 19.0	203.7 ± 54.3	152.8 ± 11.9	140.0 ± 3.9	91.7 ± 8.2	88.5 ± 5.0	135.6 ± 28.1	140.4 ± 25.4
	Acetic acid	% VS	57.0 ± 6.3	44.2 ± 3.7	44.6 ± 2.3	39.4 ± 2.7	43.7 ± 5.9	45.7 ± 2.1	38.5 ± 6.9	38.4 ± 12.4	22.4 ± 4.8	25.7 ± 5.1	42.1 ± 4.0
	Propionic acid	% VS	1.71 ± 0.30	1.18 ± 0.08	1.98 ± 0.50	1.75 ± 0.28	1.45 ± 0.32	1.74 ± 0.07	0.37 ± 0.19	0.75 ± 0.16	0.63 ± 0.19	0.88 ± 0.26	5.16 ± 2.04
	Isobutyric acid	% VS	0.78 ± 0.12	0.60 ± 0.04	1.13 ± 0.30	1.08 ± 0.18	0.75 ± 0.12	0.93 ± 0.07	0.62 ± 0.14	1.04 ± 0.33	1.19 ± 0.41	1.61 ± 0.35	1.95 ± 0.19
	Butyric acid	% VS	10.6 ± 2.8	20.0 ± 2.3	21.7 ± 2.0	23.5 ± 2.7	23.5 ± 3.5	18.9 ± 0.8	20.5 ± 4.1	20.9 ± 5.4	38.2 ± 8.3	27.8 ± 3.8	17.9 ± 3.2
	Isovaleric acid	% VS	1.70 ± 0.45	1.46 ± 0.30	2.43 ± 0.43	2.30 ± 0.53	1.15 ± 0.29	1.69 ± 0.14	1.23 ± 0.22	2.62 ± 0.78	2.71 ± 1.22	4.03 ± 0.98	4.14 ± 0.42
	Valeric acid	% VS	0.78 ± 0.13	0.61 ± 0.05	1.21 ± 0.41	1.26 ± 0.19	0.89 ± 0.14	1.00 ± 0.05	0.45 ± 0.23	0.51 ± 0.16	0.76 ± 0.08	0.95 ± 0.27	3.97 ± 1.42
FERMENTER C	pH	-	5.66 ± 0.36	5.83 ± 0.30	6.19 ± 0.17	6.02 ± 0.12	6.00 ± 0.11	6.16 ± 0.20	6.81 ± 0.42	7.07 ± 0.06	6.82 ± 0.13	6.99 ± 0.11	6.96 ± 0.19
	VFA	g/L	5.54 ± 0.71	7.17 ± 1.13	10.2 ± 0.6	10.6 ± 0.7	7.25 ± 1.80	5.68 ± 0.65	6.40 ± 1.49	11.0 ± 1.0	11.6 ± 0.7	15.3 ± 2.1	10.7 ± 1.5
	sCOD	g/L	46.2 ± 6.0	48.4 ± 2.5	47.4 ± 5.4	51.7 ± 6.8	48.6 ± 8.2	48.5 ± 2.8	52.2 ± 12.1	60.0 ± 3.5	43.8 ± 1.4	59.3 ± 18.4	44.5 ± 1.2
	COD_VFA_/sCOD	%	13.6 ± 2.2	13.4 ± 2.6	21.8 ± 3.1	21.0 ± 3.9	15.6 ± 3.4	10.8 ± 0.6	13.3 ± 4.5	19.2 ± 2.1	27.1 ± 1.7	27.1 ± 6.0	23.1 ± 2.2
	NH_4_^+^-N	g NH_4_^+^-N/L	0.63 ± 0.28	0.50 ± 0.03	0.77 ±0.19	0.55 ± 0.33	0.27 ± 0.06	0.33 ± 0.08	-	0.74 ± 0.29	1.12 ± 0.01	1.46 ± 0.19	1.56 ± 0.09
	VFA yield	mgVFA_eff_/gVS_inf_	101.6 ± 25.9	118.7 ± 21.5	179.0 ± 12.4	195.1 ± 9.8	205.8 ± 59.3	141.9 ± 13.2	190.0 ± 13.6	180.7 ± 15.4	202.9 ± 6.2	250.6 ± 41.6	199.4 ± 39.0
	Acetic acid	% VS	57.0 ± 7.6	58.6 ± 4.2	49.3 ± 4.4	41.9 ± 3.9	42.6 ± 3.4	43.1 ± 1.3	49.6 ± 4.7	53.0 ± 1.7	53.7 ± 1.2	50.2 ± 2.9	48.7 ± 3.0
	Propionic acid	% VS	1.79 ± 0.28	1.26 ± 0.17	1.77 ± 0.60	1.61 ± 0.31	1.52 ± 0.34	1.78 ± 0.06	0.79 ± 0.32	7.33 ± 1.72	7.52 ± 0.7	7.69 ± 1.48	4.62 ± 0.94
	Isobutyric acid	% VS	0.79 ± 0.13	0.73 ± 0.10	1.10 ± 0.37	1.05 ± 0.19	0.72 ± 0.11	0.87 ± 0.08	0.69 ± 0.05	1.12 ± 0.18	1.29 ± 0.19	1.71 ± 0.91	1.67 ± 0.19
	Butyric acid	% VS	10.1 ± 2.5	9.4 ± 1.8	19.5 ± 3.3	23.1 ± 3.3	24.8 ± 2.5	18.0 ± 2.1	20.4 ± 1.3	17.7 ± 1.3	17.1 ± 0.5	17.2 ± 1.6	18.4 ± 2.8
	Isovaleric acid	% VS	1.72 ± 0.45	1.77 ± 0.33	2.30 ± 0.45	2.12 ± 0.29	1.10 ± 0.26	1.69 ± 0.15	1.19 ± 0.31	1.66 ± 0.4	2.01 ± 0.32	2.63 ± 0.49	3.44 ± 0.65
	Valeric acid	% VS	0.81 ± 0.13	0.56 ± 0.08	1.03 ± 0.40	1.09 ± 0.21	0.93 ± 0.13	1.00 ± 0.04	0.64 ± 0.17	3.39 ± 2.02	5.32 ± 0.37	5.58 ± 0.67	4.63 ± 1.49

**Table 4:** Main characteristics of the batch tests of food waste using mature compost at a pH of 7.

	Units	0%	2.5%	3.5%	4.5%	FW Only	Compost Only
Food Waste weight	g	44.50	43.39	42.95	42.50	200	-
% VS of Food Waste in the mixture	%	50	43	41	39	100	-
Compost weight	g	-	5	7	9	-	9
% VS of Compost in the mixture	%	-	13	18	22	-	100
Inoculum weight	g	155.50	151.61	150.05	148.50	-	-
% VS of Inoculum in the mixture	%	50	43	41	39	-	-
Initial VS content	%	2.32	2.65	2.89	2.97	9.59	0.40
Initial soluble COD	g COD/L	40.56	39.54	39.14	38.73	35.61	6.53
Final soluble COD	g COD/L	48.47	47.88	49.06	42.93	n.a	6.86
Initial NH_4_^+^-N	mg NH_4_^+^-N/L	728	734	727	680	n.a	7
Final NH_4_^+^-N	mg NH_4_^+^-N/L	1164	1635	1724	1803	n.a.	195
VFA Concentration and Distribution at Day 10							
VFA concentration	g COD/L	12.94	13.28	13.43	11.74	5.18	0.23
Acetic Acid	% COD	37.5	37.4	38.2	35.9	76.2	53.0
Propionic Acid	% COD	6.8	7.3	7.5	7.3	13.5	-
Isobutyric Acid	% COD	2.6	2.5	2.6	2.6	2.9	-
Butyric Acid	% COD	18.4	19.6	19.4	19.4	1.2	22.0
Isovaleric Acid	% COD	5.7	5.3	5.2	5.4	1.1	-
Valeric Acid	% COD	1.3	1.4	1.4	1.5	1.6	-
Isocaproic Acid	% COD	0.1	0.1	0.1	0.1	0.4	-
Hexanoic Acid	% COD	25.7	24.8	24.3	26.3	1.6	24.9
Heptanoic Acid	% COD	1.7	1.6	1.4	1.5	1.5	-

n.a.: Not analyzed.

**Table 5:** Main characteristics of Food Waste from a university canteen in each collection period.

	Units	FW1	FW2	FW3	FW4	FW5	FW6	FW7	FW8	FW9	FW10	FW11	Range
Period	Days	1–20	21–40	41–57	58–72	73–96	97–114	115–132	133–145	146-155	156–180	181–198	-
pH	-	4.33 ± 0.61	6.54 ± 0.34	6.62 ± 0.75	6.46 ± 0.79	6.65 ± 0.23	6.67 ± 0.42	7.09 ± 0.40	6.11 ± 1.49	6.96 ± 1.15	5.73 ± 1.28	5.17 ± 0.85	4.33–7.09
Total Solids (TS)	% w/w	5.88 ± 1.63	7.31 ± 1.10	7.27 ± 0.35	6.57 ± 0.52	4.76 ± 0.38	4.45 ± 0.15	4.71 ± 0.42	7.03 ± 0.44	7.57 ± 0.96	6.39 ± 0.56	6.10 ± 1.23	4.45–7.57
Volatile Solids (VS)	% w/w	5.51 ± 1.68	6.12 ± 0.75	5.71 ± 0.24	5.46 ± 0.43	3.83 ± 0.24	3.78 ± 0.26	3.90 ± 0.28	6.40 ± 0.84	6.43 ± 1.03	5.79 ± 0.45	5.64 ± 1.18	3.78–6.40
VFA	g/L	1.08 ± 0.16	1.03 ± 0.08	1.21 ± 0.07	1.49 ± 0.18	0.98 ± 0.10	1.32 ± 0.13	0.93 ± 0.14	0.95 ± 0.28	0.75 ± 0.15	0.72 ± 0.19	0.83 ± 0.11	0.72–1.49
Soluble COD (sCOD)	g/L	40.6 ± 5.6	37.0 ± 2.9	38.7 ± 4.3	32.3 ± 14.7	31.1 ± 0.7	45.4 ± 1.6	51.0 ± 4.7	51.8 ± 4.7	14.9 ± 3.4	35.7 ± 21.8	31.9 ± 5.4	14.9–51.8
NH_4_^+^-N	mg NH_4_^+^-N/L	14.2 ± 3.9	34.1 ± 4.8	50.5 ± 1.3	26.7 ± 6.2	25.1 ± 7.1	13.0 ± 1.0	27.4	32.2 ± 12.0	26.6 ± 6.9	79.5 ± 23.8	107 ± 10	13–107
